# Barriers, enablers and motivators of the “I’m an active Hero” physical activity intervention for preschool children: a qualitative study

**DOI:** 10.3389/fped.2024.1333173

**Published:** 2024-01-31

**Authors:** Mosfer A. Al-walah, Michael Donnelly, Neil Heron

**Affiliations:** ^1^ Centre for Public Health, School of Medicine, Dentistry and Biomedical Sciences, Queen’s University Belfast, Belfast, United Kingdom; ^2^ Department of Physical Therapy, College of Applied Medical Sciences, Taif University, Taif, Saudi Arabia; ^3^ School of Medicine, Keele University, Newcastle-Under-Lyme, Staffordshire, England

**Keywords:** bold physical activity programme development and implementation, health promotion, preschool children, obesity prevention, qualitative study

## Abstract

**Background:**

Insufficient physical activity (PA) in early childhood is linked to adverse health outcomes and a heightened risk of obesity. Successful PA programmes often require input from key stakeholders, such as parents and educators. However, research on stakeholders’ perspectives regarding PA programmes for preschool children is limited, impeding effective programme design and implementation.

**Objectives:**

This study aims to explore the perspectives of key stakeholders to gain insights into the challenges, facilitators, and motivators that influence the planning, execution, and sustainability of the “I'm an Active Hero (IAAH) intervention component,” a preschool-based initiative designed to promote PA among young children.

**Methods:**

Semi-structured interviews were conducted in Saudi Arabia with individual preschool principals (*n* = 2), and focus group discussions were held, respectively, with preschool staff members (*n* = 4, all female) and parents (4 mothers, 5 fathers).

**Results:**

A thematic analysis identified four main themes: (1) Barriers to parental involvement in preschool PA interventions, such as time constraints, lack of flexibility, limited space, and a shortage of trained staff; (2) Risks and benefits of children's programme participation; (3) Motivators including rewards, non-financial incentives, and concerns about childhood obesity and a sedentary lifestyle; (4) Facilitating factors for overcoming barriers, including staff training, time reallocation, staff coordination, space optimization, non-financial incentives, and sustaining partnerships.

**Conclusion:**

This study's findings are crucial for childcare professionals, preschools, education authorities, and policymakers, offering valuable insights for future research. However, further collaboration with key stakeholders is essential to enhance individual attitudes and preschool policies for effective intervention implementation.

## 1 Introduction

Physical activity (PA) is crucial for promoting health and well-being in young children. Regular PA in early childhood is associated with a multitude of benefits (1). Recent research underscores the significance of PA during these formative years for optimal health. Studies indicate a positive correlation between increased PA and favourable outcomes such as bone density, enhanced cardiovascular health, better body composition, cognitive development, prevention of childhood obesity and the advancement of motor skills (2, 3). Moreover, the early childhood phase isa critical period for establishing lasting healthy behaviour patterns, which may continue into middle childhood and early adulthood (4, 5).

Despite evidence highlighting the importance of sufficient PA in this age group, recent studies reveal worryingly low activity levels in young children, with some assessments indicating that only around 4% of waking time spent being active (5, 6). Assuming a 13-hour waking day for a young child (7), this is markedly below international guidelines recommending 180 min of light, moderate, or vigorous-intensity PA (LMVPA) per day for preschoolers (8–10). Leading health organisations like the World Health Organization emphasise the urgency of feasible PA initiatives to foster activity in young children (11). Consequently, interventions aimed at increasing increase PA have gained prominence. However, due to the complexity of human behaviour, tailored approaches are necessary. Recognising the critical role of PA in the early years, various countries worldwide have implemented interventions and programmes aimed at promoting PA among preschool-aged children. However, the effectiveness of these interventions has varied (12–14).

Recent studies have demonstrated that the majority of interventions aimed at increasing PA in young children are implemented primarily in developed countries (15, 16), This highlights the critical need to focus on young children in developing regions, where there is a noticeable lack of randomised trials, particularly in early childhood (17, 18). This issue is intensified by alarmingly high obesity rates in these countries, such as those in the Gulf Cooperation Council (GCC), which have some of the highest obesity rates globally (19). Contributing to this problem are low PA levels, shaped by the distinct social, cultural, and environmental factors in these regions (19).

The distinct cultural, social, and environmental contexts within GCC nations present substantial challenges to the enhancement of PA levels. Factors such as rapid urbanisation and extreme summer temperatures, which often exceed 40°C, act as considerable obstacles to outdoor physical activities (19). The limited research tailored to these specific regions has resulted in an inadequate understanding of the strategies required to implement successful and effective PA interventions. While it may be useful to adapt and evaluate successful PA interventions from developed countries for implementation in developing country contexts while respecting cultures and contexts, evidence indicates that interventions primarily focused on developed countries’ contexts may not be directly applicable to the geographic and cultural landscapes of developing nations (20). Barriers to PA in these nations are expected to be markedly different from those in, for example, Western environments (20).

In Saudi Arabia, the rising prevalence of childhood obesity has rendered the promotion of PA among preschool children a critical public health priority. Given the notable dearth of interventions targeting this age group (21), highlighting the need to involve key stakeholders such as parents, preschool staff, and the children themselves in in creating environments that encourage PA (22). To address these research gaps, we have developed and designed the “I'm an Active Hero”(IAAH) programme.

The IAAH programme is a comprehensive 10-week preschool-based behaviour change initiative designed to promote PA and reduce sedentary behaviour among 3-5-year-old children, with family involvement. Developed based on the principles of the Medical Research Council's (MRC) Framework for the Development and Evaluation of Complex Interventions (23, 24), the programme underwent a systematic development process, involving key steps, such as a thorough systematic review to identify effective behaviour change techniques and input from experts in the field. Preschool teachers facilitated the intervention, undergoing two preparatory sessions led by the lead researcher to ensure consistent and effective delivery. The face-to-face delivery method was chosen based on prior research indicating its effectiveness (25, 26). The IAAH programme focuses on two crucial aspects of energy balance: increasing PA and reducing sedentary time. Intervention materials were designed for use both at preschool and at home, employing various strategies such as setting environmental changes, structured PA sessions, and classroom movement breaks. Moreover, educational materials and interactive activities were provided for parents and caregivers at home. Detailed information about the specific content and activities included in the IAAH programme will be presented in a separate publication to provide a comprehensive understanding of the intervention's components.

Most of the existing literature predominantly centres on participants’ experiences post-study conclusion. However, the generalisability of these findings might be constrained, given that they pertain to a singular programme within a distinct context (27). While the value of engaging key stakeholders such as parents and teachers in designing and implementing PA programmes is recognised by (28), research indicates limited capture of their perspectives regarding PA interventions specifically (29). Understanding these stakeholders’ viewpoints, experiences, and perceptions is essential for the effective design and implementation of PA interventions in preschool settings (30). Additionally, gaining insights into barriers, enablers, and motivators for participation can facilitate tailoring interventions to meet context-specific needs and preferences.

Traditionally, involving key stakeholders facilitates the successful launch and scale-up of population-wide initiatives, as they may contribute to and inform programme design and delivery (28). Parental and caregiver roles significantly shape behaviour change in preschoolers (30, 31). Evidence suggests engaging practitioners and parents is key to childhood obesity prevention interventions. Consulting these stakeholders can provide valuable insights into their potential contributions and programme implementation capacity (31, 32). Moreover, they can offer strategies and actions, including suggested modifications, to ensure the adaptability and sustainability of proposed programmes in real-world contexts (32). Unfortunately, researchers often overlook this phase, resulting in limited evidence reflecting stakeholder viewpoints to inform the expansion and implementation of preschool-based or home-based PA initiatives, especially those promoting activity in young children (29). Researchers have highlighted the need to ensure interventions meet the target population's needs and capitalise on their strengths, involving strategies to help parents modify children's behaviours (33). While parents may know ideal PA practices, practical implementation can be challenging (32, 34, 35). Although some obesity prevention efforts focus on primary schools (12, 36), preschool environments often lack such initiatives (14, 15). Before introducing behaviour change programmes, it is crucial to thoroughly understand the perspectives and awareness of preschool staff and parents regarding daily routines, best achieved through qualitative research (29, 37).

Complex interventions typically comprise multiple interconnected elements and find extensive application within health services, public health, and social policy interventions (38). The MRC has been widely adopted for elucidating the mechanisms underlying multifaceted interventions (39). It examines how context influences intervention outcomes and sustainability. This framework provides a systematic, iterative approach to the design, assessment, and implementation of complex programmes, including those in public health and behavioural domains (39). Central to this framework are distinct phases underscoring the need to understand real-world contexts, foster adaptability, and consistently refine strategies through feedback. This model emphasises evidence-based and theoretical guidance during development, feasibility studies and pilot testing, comprehensive evaluations of effectiveness and cost-effectiveness, and strategic planning for widespread dissemination, monitoring, and long-term follow-up (39). A key feature is the persistent emphasis on substantive stakeholder engagement, ensuring feasibility, acceptability, tailoring, and sustainability within specific contexts (39, 40). Stakeholder viewpoints are instrumental in iteratively refining interventions. Essentially, this framework underscores the paramount importance of deep-rooted stakeholder collaboration in developing, evaluating, and implementing comprehensive public health interventions, a cornerstone for the successful translation of evidence-based programmes into meaningful practices (40). However, literature elucidating key stakeholder viewpoints to inform PA programme design and implementation for young children is scarce (29). This study aimed to explore key stakeholder perspectives on the IAAH programme in Saudi preschools, specifically gaining insights into design, implementation, facilitating factors, motivational drivers, and barriers. The goal was to gather insights to shape future implementation and expansion in real-world settings.

## 2 Materials and methods

A qualitative research paradigm was deemed the appropriate investigative approach for addressing the research topic (41) and to eliciting stakeholder perspectives on the design and delivery of the IAAH programme in preschools in Saudi Arabia (42).This approach entailed conducting focus groups with relevant stakeholders (teachers and parents) as well as individual interviews with principals. The data collected were analysed using thematic analysis (43, 44).

Employing both individual in-depth interviews and focus group interviews within a qualitative study provides complementary strengths, yielding robust insights and rich, multi-faceted data. This approach capitalises on the unique advantages of each technique (45). Stokes (2006) elucidates that individual interviews allow a researcher to deeply explore personal perspectives and meanings, whereas focus groups grant access to group norms and dynamics (46) Combining these techniques enables triangulation by comparing and contrasting findings, thereby enhancing the credibility and completeness of the qualitative inquiry (47). Thus, leveraging the distinct benefits of individual and group interviews is advantageous for producing comprehensive and multi-faceted insights in qualitative research.

The formative nature of the research approach was integral to a stakeholder engagement strategy. This strategy involved the parents of preschool PA programme “users” and school leaders in contributing to the planning of the intervention or programme's scale-up. Such involvement is pivotal in facilitating programme uptake in real-world settings (48). It is recognised that delivering a programme at scale within existing service and policy contexts will require practical considerations and may pose potential challenge (48).

The need for methodological reflexivity was acknowledged, with the researchers considering themselves integral to the knowledge-generation process. The Standards for Reporting Qualitative Research (SRQR) (49) (see Supplementary File S1) guided both the methodological conduct of the qualitative study and its reporting and presentation in this paper.

### 2.1 Study design

This study employed a qualitative design to explore stakeholder perspectives regarding the barriers, enablers and motivators associated with the preschool based IAAH programme and its design and delivery. The study was conducted at Saudi Arabian preschools between December 2022 and February 2023.

### 2.2 Participants and recruitment

After obtaining ethical approval and participants consent, the study proceeded with qualified preschool staff, comprising teachers, classroom assistants and principals, from both public and private preschool centres in Taif, Saudi Arabia. Parents of preschool children aged 3–5 were also included as participants. A purposive sampling approach was employed, intentionally selecting participants and sampling units based on specific traits, consistent with the study's objectives.

### 2.3 Focus groups and interviews

Focus groups were conducted separately with parents and teachers/classroom assistants. This method was chosen due to its frequent use in studies of programme design, development, and formative evaluation, as it is a tried, tested, and efficient way of eliciting views, beliefs, and experiences from multiple participants or stakeholders simultaneously in the same setting. Three focus groups involving 15 participants were conducted across preschools in Taif, Saudi Arabia. Specifically, two focus groups were held with parents (*n* = 9), and one focus group involved teachers/classroom assistants (*n* = 4). Additionally, a one-to-one interview was conducted separately with two principals of preschools in this area.

### 2.4 Interviews with principals

The interviews with principals were held in person in their offices at preschools. Informed consent was obtained from the principals before commencing the interviews, which were audio-recorded using a Dictaphone, lasted approximately 25–35 min, and transcribed verbatim. The semi-structured interview format allowed flexibility to explore various aspects of the programme, including its components, delivery method and potential organisational revisions, all grounded in a comprehensive literature review (see Supplementary File S2).

### 2.5 Parents’ focus group

Preschool staff extended written invitations to all parents (*n* = 42) for participation in designated focus group sessions. Despite these efforts, securing attendance faced challenges due to time limitations, privacy concerns, and general disinterest among some parents. A total of 14 parents consented to participate in these sessions. Ultimately, only 9 individuals were able to attend the scheduled sessions. Holding gender-segregated focus groups for mothers (*n* = 4) and fathers (*n* = 5), each group had an average of approximately five participants, aligned with cultural expectations and legal mandates in Saudi Arabia that prohibit mixed-gender interactions during interviews. To facilitate effective data gathering, internet-mediated communication via Zoom® was employed for the focus groups. Participants were provided with comprehensive information and consent forms. Additionally, the sessions were audio-recorded using a Dictaphone and lasted a duration of 45–60 min. The focus groups followed a topic guide (see Supplementary File S3).

### 2.6 Focus groups: teachers/classroom assistants

In two preschools, all teachers and classroom assistants (nine in total) received invitations to join focus group sessions. Six out of the nine invited consented, but only four (three teachers and one assistant teacher) managed to attend. Reasons cited for non-participation included time constraints and conflicts with other teaching and administrative responsibilities. These focus groups, which had an average of four participants, were conducted in the preschool staff room. Similar to the parent groups, the sessions aimed to explore factors influencing the implementation of the IAAH program in the preschool context. The participants were briefed with information and consent forms before the discussions. These sessions, also approximately 45 min in duration, were audio-recorded and followed a structured topic guide (see Supplementary File S4 for further details).

### 2.7 Data management and analysis

A rigorous and systematic approach was employed to quality assure the credibility and dependability of the study's findings. The group and individual interviews were recorded and transcribed verbatim, forming the foundational data for analysis. To establish the research's trustworthiness, a multifaceted strategy was implemented, drawing on established methods (42). This strategy included transparent documentation of the data collection, interpretation, and analytical processes, facilitating potential replication by other researchers.

The thematic analysis in this study adhered to the guidelines proposed by Braun and Clarke (43, 44). Key themes, subthemes and categories were identified through immersion in the data and inductive coding. The analysis involved theme identification and compilation of results. To ensure rigour, intercoder reliability procedures were implemented. A subset consisting of 30% of the interview and focus group transcripts were independently coded by two researchers (M.A. and N.H.). Minor terminological differences in codes were resolved through in-depth discussion and consensus between the coders. Coding consistency was assessed using Cohen's kappa statistic (*κ*), with *κ* ≥ 0.70 indicating acceptable agreement (50). The kappa values were 0.82 for interviews and 0.76 for focus groups, indicating substantial agreement between coders. All research team members (M.A., N.H., and M.D.) reviewed, refined, discussed, and named the developing codes and themes. If sample quotes are included to demonstrate key themes, the coder(s) who derived each specific quote will be indicated to further enhance confirmability.

Reaching data saturation is essential in qualitative research. Saunders et al. (51) explain, there is no single point where saturation is achieved across all studies. To evaluate if saturation was being reached in this study, emerging themes were systematically identified during data analysis. Saturation was determined when additional data collection and review revealed no new substantive themes (52). This iterative approach balances gathering sufficiently rich data with pragmatic constraints.

Hennink and Kaiser (2022), in their recent systematic review, highlight the importance of prioritizing data adequacy over participant numbers in qualitative research. An effective sample size should ensure a comprehensive and accurate representation of the studied phenomenon. Typically, saturation in focus group discussions occurs within a range of 4–8 groups, but the studies in the review showed variations from 2 to 40 groups (53). More homogeneous populations and narrowly defined research goals required fewer groups to reach saturation, whereas diverse samples and broader objectives necessitated larger sample sizes (53). Therefore, sample sizes, like the focus groups in our study, that fall within this range and employ homogeneous samples and specific objectives can be effective, especially when the focus is on data depth and richness rather than sheer quantity. However, determining and justifying sample sizes in qualitative research must consider various study characteristics, including study objectives, the complexity of the phenomenon, instrument structure, and sampling strategy (54). Factors such as sample stratification, the researcher's qualitative research expertise, saturation goals, and the desired degree of saturation also play a significant role in making this determination (54).

### 2.8 Ethics statement

Ethical approval for the study was obtained from the Saudi Arabian]Ministry of Health's Research and Studies Department (IRB Registration Number with KACST, KSA: HAP-02-T-067). All ethical standards and regulations were adhered to throughout the study, including obtaining informed consent from all participants and ensuring their privacy and confidentiality.

## 3 Results

Of the 40 preschool centres contacted, two preschool principals consented for their centres to participate, and contact details for interested staff were provided. Ultimately, six out of nine staff members agreed to participate, with four attending the discussion group (Table 1). Parents were informed about the study through preschool staff, and interested individuals were contacted via telephone. Forty-two invitations were distributed of which 14 returned with interest to participate and only 9 individuals (four mothers and five fathers) agreed to participate after the discussions (Table 2). Both preschool principals were female, one with an undergraduate degree and five years of experience, and the other with a postgraduate degree in childhood education and more than ten years of experience.

**Table 1:** Socio-demographic profile of participating teachers.

Characteristics		Number (*n* = 4)
Gender	Female	4
Degree type	Education	1
	Childhood education	3
Qualifications (education level)	Undergraduate	3
	Postgraduate	1
Preschool work experience	5–9 years	1
	10–14 years	2
	15 or more years	1
Role	Teachers	3
	Assistant teachers	1
Age group	26–35	1
	36–45	1
	46–55	2

**Table 2:** Demographic characteristics of participating parents.

Demographic characteristics	Total (*n* = 9)
Sex	
Female	4
Male	5
Marital status	
Married (men)	5
Married (women)	3
Separated (women)	1
Age group	
26–35	1
36–45	2
46–55	1
26–35	2
36–45	1
46–55	1
Over 55	1
Educational level	
University or more	8
High school or diploma	1

**Figure 1:**
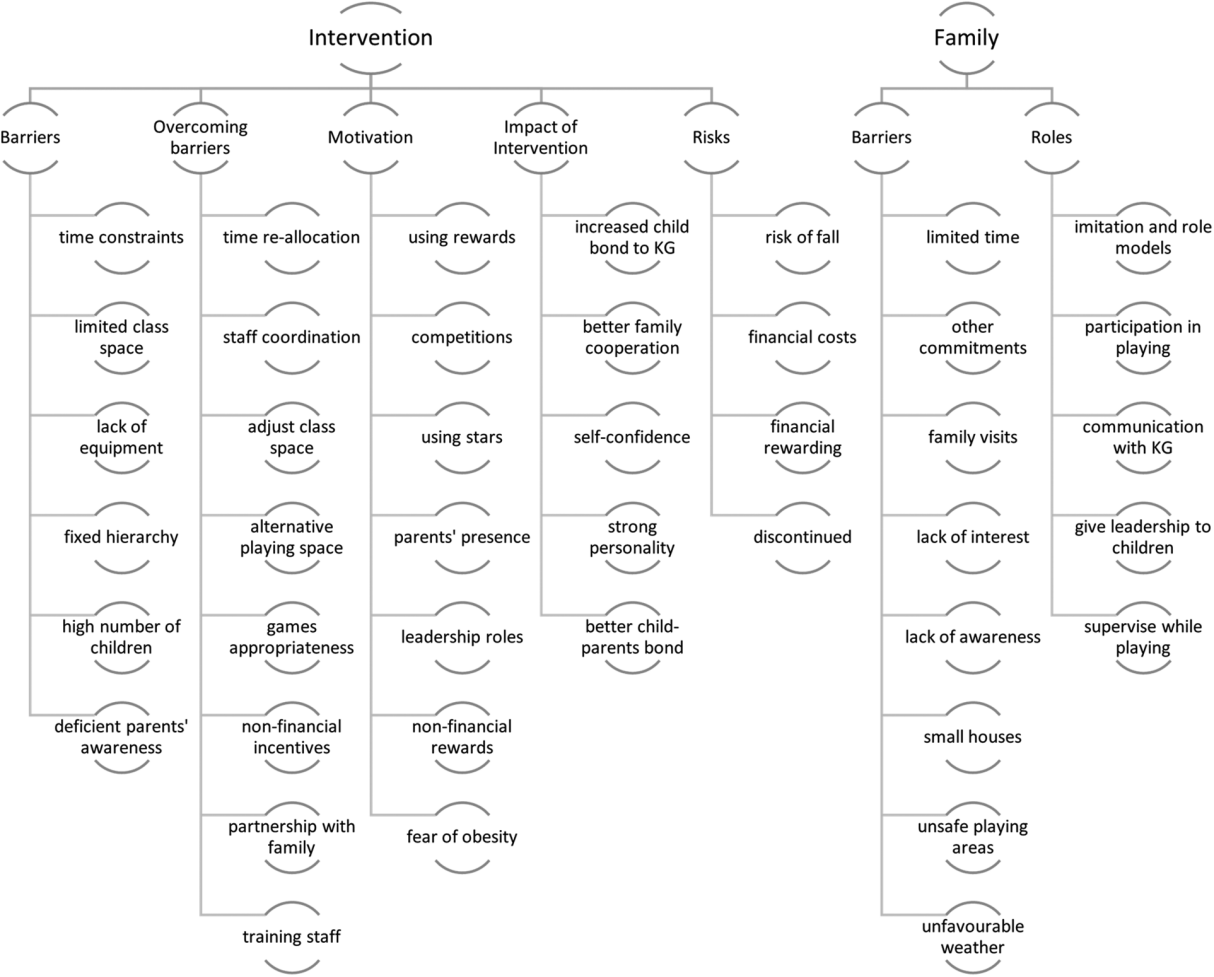
Conceptual mapping of the main themes and subthemes, KG, kindergarten.

The interpretation and thematic analysis of the interview data revealed several overlapping themes (Figure 1); therefore, the results from staff and parents are presented and discussed together. However, for simplicity and clarity, we categorised the themes into two main categories: family-related (two themes) and intervention-related (five themes). Each of the main themes is summarised below, supported by examples of relevant quotes from the interviews and focus groups.

Overall, principals, parents, and teachers welcomed the programme, commended its potential benefits, and expressed enthusiasm about having it implemented with the children under their care.

“I emphasise its importance and I see it as an easy programme. It will be easy to apply. One of the most important factors is that it is inexpensive.’’ (Principal 1)

The teachers and the parents also shared their views on the perceived risks and barriers and how to overcome them “Yes, it does exist. We have some obstacles to that, such as narrow classes and a large number of students.’’ (Teacher 2)

“First, not having enough time. Secondly, the lack of awareness of such beautiful and useful programmes. Sometimes the lack of resources. This means that I see that the three factors are among the most important three factors that could be a barrier or hindrance to the implementation of such programmes.” (Parent 3)

“Also, one of the barriers that prevent my child from practising activities is the lack of time—psychological conditions, like work and so on, like the fear of falling and injuries. That's it.” (Parent 4)

### 3.1 Intervention-related themes

#### 3.1.1 Perceived barriers

The teachers’ perceived barriers were mostly related to logistic and managerial issues. For example, they raised concerns about the lack of time available for any additional activities and the lack of flexibility in their timetables. They mentioned that the curriculum set by the Ministry of Education made it difficult to have the flexibility necessary to include the programme. They also described a lack of adequate space, especially with the growing number of children joining the preschool settings. The parents in the focus groups also discussed barriers such as limited time due to family commitments and responsibilities, a lack of awareness about the importance of PA for preschoolers, small house sizes that limit active play, and unfavourable weather conditions. Some parents emphasized the need for more education and research to increase awareness and knowledge about PA for young children.

“I expect that what can hinder PA [include], for example, you have time parameters. Are they committed to a predetermined academic timetable? Is there also a limitation in classroom space, or …? The number of students has also increased.” (Teacher 4)

“I summarise my view in two points. The impediments depend on the family, the lack of comprehension from the family and the lack of trained staff in an integrated manner to guide the child to this point.” (Teacher 2)

“First, not having enough time. Secondly, the lack of awareness of such beautiful and useful programmes. Sometimes the lack of resources. This means that I see that the three factors are among the most important three factors that could be a barrier or hindrance to the implementation of such programmes.” (Parent 5)

“I expect the biggest obstacle in principle is … the culture of the father and mother. Not all parents are on the same level of culture and awareness. You find … for example, a mother who is an employee, or a working father … Generally, it all returns to the father and mother … if they are not interested or are busy, or they are not convinced of the idea in the first place. Here is the problem.” (Parent 2)

Moreover, both the teachers and parents expressed a general emphasis on the importance of family involvement for the programme to be successful.

“About the involvement of parents, we know that children by their nature like to imitate their parents. For example, you see a boy who likes to imitate his father when he sees him talking or making a phone call.” (Teacher 3)

#### 3.1.2 Perceived risks

Perceived risks were mostly associated with the actual application of the programme. These included the following:

1. Risk of falls

Both parents and teachers expressed concerns about the risk of children falling and injuring themselves during PA. Notably, mothers exhibited greater concern regarding this risk.

“As a mother, I have observed that children in kindergarten are at risk…they are prone to falling unless they possess a good understanding (perception). If engaging in sports is necessary, both the teacher and the mother should be present.” (Parent 3)

2. Financial costs

The financial cost was of greater concern to the preschool staff, who associated this cost with the risk of programme discontinuation.

“Just as the programme is engagingly simplified, it should also be financially viable. We should try to replicate the simplicity in the games to make it affordable in terms of material cost, which should not be above the reasonable financial capacity of the preschool.” (Teacher 4)

3. Unsustainability of reliance on financial rewards and discontinuation

It was suggested that motivation through financial or other tangible rewards is unsustainable, leading to the risk of discontinuation if it imposes too much financial burden on families.

“Most families can't reward [their children] for everything. And we fear that this will be a habit if the child says, “If you give me a reward, I will work and be active, and if you don't, I will not be active”. I expect … that motivation can be given in another way, so that there are no expensive prizes that are burdensome on the parents, such as giving the spirit of leadership.” (Parent 4)

#### 3.1.3 Perceived benefits

There was consensus that the IAAH programme would be beneficial for children, and there were complementary views among teachers and parents. The perceived benefits include:

1. Strengthening children’s bodies (bones and muscles) and personalities

The IAAH programme and PA in general are perceived as beneficial for children’s physical and emotional development.

“PA is very important for children in this age group because it helps them strengthen their muscles and helps them in early activity. If we are motivated and aware of encouraging sports participation in kindergarten, the child will develop a love for the sport, and it will be something essential in their life.” (Parent 3)

“I am raising a strong child with a strong personality and an intrepid personality who presents without fear during participation and competitions, especially with family involvement.” (Parent 2)

2. Enhancing children's sense of leadership

Parents expressed views on their children's sense of leadership in terms of intangible motivation and as an anticipated outcome of the programme, sharing personal experiences of how they nurtured it with their children.

“Instil in the child the spirit of leadership; for instance, they might be told, ‘Today, you will lead us in a game. Choose a game, gather us together, and facilitate the game.’’ (Parent 4)

3. Boosting the bond between children and their parents

In addition to the physical and psychological aspects, there was a focus on the programme's impact on familial relationships between parents and children.

“From my point of view, the involvement of parents in this programme with their children is very important, as it will strengthen the child's personality, encourage the child to participate … and strengthen the child's bond with their parents.” (Teacher 2)

4. Improving the partnership between the parents and the kindergarten

“I think that the I Am an Active programme will give my son a boost in his self-confidence and also enhance his skills and inclinations, increasing cooperation between kindergarten and home, promoting family involvement, and also teach about the skills and tendencies of my child.” (Parent 1)

#### 3.1.3 Motivating and enhancing factors

Both parents and teachers identified numerous motivating factors contribute to the success of the IAAH programme, mainly through the motivation of the children themselves. These factors emerged from shared experiences and descriptions of methods used to motivate the children. These suggestions can be summarised as follows:

1. Using tangible rewards to motivate children, like toys, attending a special event, etc.
2. Conducting competitions among the children in kindergarten and at home.
3. Using stars (or other stickers) whenever the child fulfils a required commitment in the programme.
4. Encouraging the presence of parents.
5. Assigning leadership roles to the children at home.
6. Applying non-financial rewards to achieve sustainability and to avoid linking the activity to tangible things only.
7. Leveraging parents’ fears of their children becoming obese or falling into a sedentary lifestyle encourages parents to be more engaged in the programme.

“Children love to be rewarded, even with simple things, and to have rewards from the things they love. I mean, for example, some children love to hear stories … we saturate them with books from which they can benefit.” (Parent 1)

“I advocate for active parental involvement. When children sense their parents’ presence, it may boost their engagement to participate. These are my personal beliefs.” (Teacher 3)

“The ideas of these games should be well-suited to the space of the house and the age of the preschool child, not above or below their age, to achieve the desired [outcome].” (Parent 2)

### 3.2 Family-related themes

Although many themes overlap between issues related to the intervention and family matters, certain issues pertain predominantly to families. For example, emphasis was placed on barriers related to limited time due to other commitments, mostly family visits and work obligations.

“We have a social life that is sacred over any other commitment. I mean, like father and mother, they are busy with whirlpools throughout the week, and after this, [the] weekend is a life of social engagement. Our social factor here is sacred over any other factor. And this could be one of the reasons.” (Parent 3)

Furthermore, there were a perceived barriers stemming from a potential lack of interest among some parents and a lack of awareness for others about the importance of PA for their children. “I say honestly, I tell you that I am surprised now that sport is important for children at this early age. I mean, I expected that sports are for older children, but the small and skinny ones also need sports! [I thought that] as long as he plays at home, then his things are fine.” (Parent 1)

Moreover, some parents emphasised the need for enhanced education and further research on PA for children.

“I urge researchers to present studies, provide awareness programmes, write to schools, write to parents who understand. Educate us so that we can apply these things at home.” (Parent 2)

Several parents, particularly mothers, highlighted that residing in small houses made indoor play unsafe, Additionally, there was mention of the impact of unfavourable weather conditions.

“Lack of awareness of families, lack of spaces in the house, lack of preparation of the place, like the building of houses to practice sports activities. I hope that every house has places designated for practising sports.” (Parent 1)

“The weather] could be hot. It could be rain; it could be cold that hinders movement … There could also be dust in the air, preventing preparation, play, or the practice of favourite hobbies.” (Parent 3)

### 3.3 Suggestions for overcoming barriers and perceived risks

Both parents and teachers shared their thoughts on potential solutions to overcome the barriers to implementation and the perceived risks of the IAAH programme. Nearly all the participants agreed that children are easy to encourage, motivate and reward. They also highlighted the need for role models, particularly parents and teachers, and emphasised the significance of parental presence during sports activities, both as supervisors and motivators. The suggestions can be summarised as follows:

1. Re-allocation of kindergarten time to specify a dedicated time for the application of the programme.
2. Coordination of staff so they can take over the programme's implementation.
3. Adjustment of classroom space, for example, by moving the furniture dedicating a specific space for the programme or providing an alternative playing space (outside the classroom).
4. Choosing appropriate games for the sex and age of the children.
5. Using non-financial incentives to encourage children to commit to the programme.
6. Enhancing and maintaining the partnership between the family and the kindergarten.
7. Training staff online and/or out-of-hours to ensure higher attendance.

“Of course, I add my voice to the voice of my colleague. The first thing focuses on two things, which are allocating a part of each class, a small part of the child's activity, and also providing sports classes. Weekly or every two to three days. This is a motivating incentive for the child to do the activity.” (Teacher 4)

“For the class, we can adjust it. We set a simplified corner in the classroom according to the teachers and their method of placing it. It is also possible to allocate it in the outdoor courtyard in the schoolyard if possible and if the place is spacious. I can implement a part of it in the kindergarten courtyard.” (Teacher 1)

“Additionally, organising competitions between families and the kindergarten can be beneficial. Providing games tailored to my child's preferences and gender is important, as activities should be appropriate and differ for boys and girls.” (Parent 3)

“For instance, at home with my children, I award a star to the child who consistently brushes their teeth. This practice fosters regularity and commitment. Similarly, we implemented this approach in “I am the hero” programme.,” (Parent 3)

“From my perspective, conducting face-to-face sessions could pose challenges due to our commitments to classes, curriculum, and tasks within the kindergarten. However, if the sessions could be organised online or remotely, or scheduled outside of working hours, I see no issue with that approach.” (Teacher 3)

## 4 Discussion

The findings of our thematic analysis shed light on the challenges and opportunities associated with implementing the IAAH programme in preschool environments in Saudi Arabia. This section places these findings in the context of current literature, highlighting their significance and implications for promoting PA among preschool children in Saudi Arabia.

### 4.1 Acceptance and enthusiasm for the IAAH programme

Our study revealed that both parents and teachers welcomed the IAAH programme, recognising its importance and expressing enthusiasm for its implementation. This positive attitude towards PA programmes in preschools is in line with previous research that underscores the value of early childhood PA interventions (55).

The enthusiasm demonstrated by both parents and teachers towards the IAAH programme correlates an increasing awareness of the necessity for holistic child development, emphasising PA in tandem with academic endeavours. Research has evidenced that such integrated approaches yield favourable outcomes in both the physical and cognitive development of children (3, 56).

### 4.2 Intervention-related themes

#### 4.2.1 Perceived barriers

One of the primary concerns identified by teachers is related to logistical and managerial issues. These challenges, such as limited time within the academic timetable and curriculum constraints, are in line with studies that have highlighted the pressures on educators to meet academic goals (57). To mitigate this, advocating for more adaptable curricular frameworks that incorporate PA might be a viable solution. The lack of trained staff in sports and a general lack of awareness about the importance of PA for preschool children reflect the need for professional development and awareness campaigns. Previous research has emphasised the importance of providing educators with appropriate training so that they can confidently deliver PA programmes (58).

Family involvement emerged as a key theme. This echoes the literature on the important role of parents in shaping children's PA behaviours (59). Encouraging family participation aligns with a holistic approach to child development that recognises the family as a central influence on children's health behaviours (60).

#### 4.2.2 Perceived risks

Concerns about the risk of children falling during PA highlight the necessity for safety measures and appropriate supervision, especially in preschool settings. Safety is a paramount consideration when designing PA programmes for young children (61, 62).

The issue of financial costs were highlighted as a concern, particularly by preschool staff. This concern resonates with the need to ensure the affordability and sustainability of PA programmes in educational settings (63). Additionally, the debate around financial incentives brings into question the long-term viability of motivational strategies.

#### 4.2.3 Perceived benefits

The recognition of the IAAH programme's benefits in strengthening children's bodies and personalities aligns with the extensive body of literature documenting the beneficial effects of PA on physical and psychological well-being (3, 64). It emphasises the importance of integrated programmes that address both physical and emotional development.

Enhancing children's sense of leadership is a noteworthy outcome identified by parents. This aligns with research emphasising the development of socio-emotional skills through PA (65). It suggests that PA programmes can contribute to broader aspects of child development.

### 4.3 Motivating and enhancing factors

The strategies proposed by parents and teachers to motivate children are consistent with the current literature on effective behaviour change techniques (66). Using tangible rewards, conducting competitions and encouraging parental involvement align with evidence-based strategies to promote PA in children (66).

### 4.4 Family-related themes

The challenges related to limited time caused by family commitments and the need for better education and research underscore the importance of engaging families in interventions aimed at young children [39]. Addressing these challenges may require family-focused strategies that consider cultural and social factors.

### 4.5 Suggestions for overcoming barriers and perceived risks

The suggestions provided by parents and teachers offer practical insights for overcoming barriers to programme implementation. Strategies such as reallocating kindergarten time and coordinating staff efforts demonstrate the importance of tailored approaches for successful programme implementation (67). Additionally, adjusting classroom space and choosing age-appropriate games aligns with established best practices for promoting PA in early childhood (58).

### 4.6 Strengths and limitations

The strength of this qualitative study lies in its in-depth exploration of a diverse group of stakeholders, including school principals, teachers, teaching assistants, and parents, who provided valuable feedback on potential barriers, facilitators, and suggestions to improve the effectiveness of PA programmes for preschool children. Furthermore, the qualitative study design allowed for a thorough and detailed examination of this previously unexplored research area. This novel approach, exemplified by the groundbreaking IAAH preschool programme in Saudi Arabia, enabled in-depth investigation of key programme aspects and the emergence of new issues through open-ended questions and inductive coding. The interviewer's intimate knowledge as the programme designer/developer, extensive experience as a paediatric physical therapist, and role as a parent of young children including preschoolers, endowed them with the nuanced understanding required to ask probing questions and provide insightful responses during interviews. While the thematic agreement was reached between authors involved in data collection/analysis, member-checking with participants was not feasible due to time constraints and the desire to avoid participant burden; this is acknowledged as a limitation. Other limitations include the small sample size (*n* = 15), which may have restricted stakeholder perspectives and ideas. There is also the possibility of self-selection bias, where more physically active participants may have been more likely to volunteer. However, this sample still provided valuable insights into overcoming barriers and the optimal strategies for implementing behaviour change programmes for preschool children.

### 4.7 Implications for future research

The exploration of the IAAH programme's implementation in various preschool contexts is a crucial area for future research. Longitudinal studies are recommended to evaluate the programme's long-term effects on children's physical and psychological well-being. Additionally, evaluating the effectiveness of different motivation strategies, including non-financial incentives, can provide valuable insights into maintaining active engagement in PA among children.

This study highlights the critical role of effective communication and the development of mutual understanding between home and school environments. This synergy is vital for the success of interventions aimed at enhancing PA levels in preschool children. Future research should therefore focus on developing strategies for preschools to engage effectively with parents and caregivers. Emphasis should be placed on fostering an environment centred around the health and well-being of the child.

Addressing prevailing perceptions about child health responsibilities and challenging deeply rooted social norms that align with health promotion are paramount. Advocating for a holistic and preventative approach to child health might be an effective strategy to overcome barriers and foster a shared understanding between homes and preschool settings. The establishment of an effective communication strategy between home and preschool is, therefore, a critical factor for the successful implementation of such programmes. Beyond their implications for the enhanced execution of the IAAH programme, our findings also hold relevance for other preschool health promotion interventions, including those focused on mental health support or weight management for young children. Incorporating primary stakeholder perspectives and ideas is a cornerstone for achieving optimal outcomes in the planning and execution of these programmes.

## 5 Conclusions

This study offers valuable insights into the IAAH programme's implementation in preschool settings, highlighting the integral role of collaborative efforts. The identification of perceived barriers, risks, benefits, and motivating factors by parents and teachers is pivotal for designing future interventions that effectively promote PA and holistic child development. Our findings highlight the urgent need of ongoing research and the importance of sustained collaboration among educators, families, and policymakers to ensure the success and evolution of such programmes.

Moreover, this study makes important contributions to the emerging field of PA programmes for preschool-aged children. The findings emphasize the significance of collaborative efforts between stakeholders in co-designing initiatives like the IAAH programme. Partnerships such as these inform the expansion and implementation of IAAH and offer key lessons for the broader research community when initiating public health interventions. Successful implementation requires integrating the program into the curriculum, aligning it with educational goals, and obtaining staff buy-in. Thus, our study's recommendations extend to policymakers, programme providers, and schools, providing comprehensive guidance for developing and executing future healthy lifestyle interventions in preschools and homes. These efforts collectively support the overarching objective of promoting PA and holistic child development within early childhood education.

## Data Availability

The raw data supporting the conclusions of this article will be made available by the authors, without undue reservation.
