# Supplementary material for: Barriers, enablers and motivators of the “I’m an active Hero” physical activity intervention for preschool children: a qualitative study
Source: Front Pediatr. 2024 Jan 31;12:1333173. doi: 10.3389/fped.2024.1333173 (PMC10864600; doi:10.3389/fped.2024.1333173)
Supplement: Supplementary file 1 [file Datasheet1.zip › Supplementary File S2 pricipals interview guides..docx]

**Supplementary file S2: Principals interview topic guide**

**The I’m an Active Hero (IAAH) Study:**

**1. General Information on Physical Activity**

1. Do you believe physical activity has benefits for pre-schoolers? Can you describe it for me? Do you think there could be any risks associated with it?
2. What type of physical activity do the children currently participate in during the preschool day?
3. Does the preschool run any kind of events that encourage students and teachers to be physically active (e.g., preschool sports day)?

**2. I’m an Active Hero (IAAH) Intervention Programme Barriers/Facilitators**

We will implement a project called I’m an Active Hero (IAAH) intervention programme, aiming to increase physical activity in young children and consist of the following:

• In preschool - Physical activity training sessions for preschool staff (2 hours each)

- increase children’s daily moderate to vigorous physical activity: introduce different class games and preschool and class modifications.

• At home – encourage parents to reduce children’s sedentary behaviours: less TV viewing, reduce electronic media use.

• At home – encourage increased family ‘active time’ such as provision of parental-child interactive homework activities and written information for parents on how to encourage their children to be active, walking, cycling, playing in the park etc

1. What are the barriers/facilitators for these ideas? Any suggestion to overcome possible barrier or enhance any facilitators?
2. Would you be prepared to support teachers and children in participating in in the I’m an Active Hero (IAAH) intervention programme during the preschool day? How?
3. How do you feel about children taking part in the I’m an Active Hero (IAAH) intervention programme during class time in preschool?
4. How do you feel children could be motivated to take part in the I’m an Active Hero (IAAH) intervention? (Incentives, competitions, rewards)
5. Do you feel there could be a change in organisational structure within the preschool in order to facilitate the I’m an Active Hero (IAAH) intervention? For example, a change in preschool environment and classes?
6. Could opportunities be offered to children to participate in I’m an Active Hero (IAAH) intervention during the preschool day and could time be allocated to this during the preschool day?
7. What is your opinion of involving parents in the I’m an Active Hero (IAAH) intervention program?
8. Preschool teachers/ classroom assistants/ will be asked to participate in two face-to-face training sessions (2 hours for each) with the principal investigator: do you think the teachers would be happy to do this? In what ways can physical activity training sessions be implemented in preschool settings, and to what extent do you think preschool staff need to be trained?
9. What do you feel would help make an I’m Active I’m an Active Hero (IAAH) intervention successful in this study?

**Any other comments?**

**Close:**

• Do you have any other thoughts or views you would like to share?

• What has it felt like to participate in a focus group? Is it what you expected? (If not, what did you expect?

**Thank participants for their time and inform them of what happens to the information.**
