# Supplementary material for: Barriers, enablers and motivators of the “I’m an active Hero” physical activity intervention for preschool children: a qualitative study
Source: Front Pediatr. 2024 Jan 31;12:1333173. doi: 10.3389/fped.2024.1333173 (PMC10864600; doi:10.3389/fped.2024.1333173)
Supplement: Supplementary file 1 [file Datasheet1.zip › Supplementary File S4 Parents focus group guides..docx]

**Supplementary file S4: Parent/caregiver focus group topic guide**

**The I’m an Active Hero (IAAH) Study:**

**1. General Information on Physical Activity**

1. Do you believe physical activity has benefits for pre-schoolers? Can you describe it for me? Do you believe there are any disadvantages?
2. What types of daily physical activities do your children engage in following preschool?

**2. I’m an Active Hero (IAAH) Intervention Programme - Barriers/Facilitators**

We will implement a project called "I’m an Active Hero (IAAH)” intervention programme, aimed at increasing physical activity in young children and consisting of the following:

- In preschool, increase children’s daily moderate to vigorous physical activity; introduce different class games; and preschool and class modifications.
- At home: The preschool will encourage parents to reduce children’s sedentary behaviours: less TV viewing, less electronic media use.
- At home – The preschool to encourage increased family ‘active time', such as provision of parental-child interactive homework activities and written information for parents on how to encourage their children to be active**,** walking, cycling, playing in the park etc.

1. What is your opinion and suggestions for these ideas?
2. What are the barriers/facilitators for these ideas?
3. How do you feel about your child taking part in the I’m an Active Hero (IAAH) intervention programme at home? What would help or hinder you as a family to do so?
4. How do you feel children could be motivated to take part in the I’m an Active Hero (IAAH) intervention? (Incentives, competitions, rewards)
5. What do you feel would help make an I’m Active I’m an Active Hero (IAAH) intervention successful in this study?

Any other comments?

**Close:**

- Do you have any other thoughts or views you would like to share?
- What has it felt like to participate in a focus group? Is it what you expected? (If not, what did you expect?)

**Thank the participants for their time and keep them updated on what happens with the information.**
